# Factors associated with trachoma in persistently endemic setting in Southern Ethiopia: a community-based cross-sectional study

**DOI:** 10.11604/pamj.2024.48.93.43242

**Published:** 2024-07-09

**Authors:** Dawit Seyum Buda, Naomi Lorrain Nkoane, Thinavhuyo Netangaheni

**Affiliations:** 1 Department of Health Studies, College of Human Sciences, University of South Africa, Pretoria, South Africa,; 2 Orbis International, Addis Ababa, Ethiopia

**Keywords:** Trachoma, neglected tropical disease, persistence, disease elimination, SAFE strategy

## Abstract

**Introduction:**

in Ethiopia, despite implementing decades-long surgery, antibiotics, facial cleanliness, and environmental improvement interventions, commonly known as the SAFE strategies, persistence and recrudescence of trachoma are common. There is limited evidence that explained the reasons. This study assesses factors associated with trachoma in persistently endemic settings.

**Methods:**

using a World Health Organization (WHO)-endorsed Global Trachoma Mapping Methodology, a two-stage cluster sampling technique was applied to select 1538 study respondents from 52 clusters. Data was collected using ODK and analysed using SPSS 28. A total of 1522 respondents were enrolled.

**Results:**

the mean age of the respondents was 33.4 and 50.5% of the respondents were females. About 32.3% (CI 30%, 34%) of the households reported the presence of at least one member of the family having one or more symptoms of trachoma. Being from poorer household (AOR=1.36, 95% CI: 1.0,1.75), presence of a household member who did not receive optimum treatment (AOR=2.8, 95% CI: 1.5, 5.2), and less than 3 doses of treatment (AOR=1.94, 95% CI: 1.32, 2.86) and presence of children ever not treated (AOR= 2.5, 95% CI: 1.5, 4.2) are associated with increased risk of manifesting symptoms of trachoma. In contrast, having optimally treated members of household (AOR=11.2,95% CI: 6.5, 19.3) and face washing with soap (AOR=0.59, 95% CI 36, 0.97) were preventive.

**Conclusion:**

trachoma is a persistent problem in the study districts. Generally, persistent, and recrudescent districts are characterised by segments of population missing optimum treatment as well as poor sanitation and hygiene practices. Our evidence supports the importance of adhering to optimal treatment guidelines, leaving no one behind, and the need for adequate treatment coverage.

## Introduction

Trachoma is the foremost infectious cause of preventable visual impairment. As of 2020, about 0.4 million people were blind and 1.6 million were visually impaired due to trachoma [1]. There is consistent evidence that trachoma-related visual impairment and blindness are declining. The encouraging progress in some countries has contributed towards reduced burden at a global scale. To date about 17 countries achieved elimination. However, progress is not uniform at the global scale. Trachoma continues to affect millions of people in 40 countries [1]. Countries and regions range from those who have eliminated trachoma centuries ago to hyperendemic countries and regions contributing to a significant proportion of the current global burden. In several cases, the variations are stark and clear within localities and subregions of the same country [2].

The World Health Organization (2023) in their progress report for 2022 estimated that in 2023, about 115.7 million people lived in the 1119 districts in which the active trachoma (TF) prevalence in children aged 1-9 years was ≥5% [3]. This marks a slight reduction from the 2022 status, at which about 125 million people lived in endemic areas that did not achieve the elimination threshold [3]. Africa bears the largest burden of population living in trachoma endemic areas, contributing to 84% of the global burden, among which 52% are in Ethiopia [3].

In 2023, trachoma was endemic in 798 districts in Ethiopia. District-based prevalence estimates show that the prevalence of active trachoma is 13%, and about 342,800 people live with trachomatous trichiasis (TT), the blinding stage of trachoma [4]. The average aggregated national level of prevalence of active trachoma declined from nearly 27% in 2015 to about 13% in 2020. In the same period, TT prevalence among adult population aged 15 years and above declined from 4% to below 1% [4].

Despite the implementation of the full SAFE strategy for several years, persistence and recrudescence of trachoma are common problems globally. Persistence indicates the inability of an intervention unit often a district, with at least two impact surveys at which TF 1-9 is ≥5%, without ever having had a TF 1-9 <5%; and recrudescence implies an intervention unit with at least one surveillance survey at which TF 1-9 is ≥5% [5]. It has been recommended to review the limitations of the current interventions, especially in hyperendemic countries and regions [2,3,6]. With the current pace of progress, delayed elimination in Ethiopia is inevitable and may further delay the global elimination of trachoma. However, the reasons contributing to persistent and recrudescence trachoma are not well documented. There are limited studies that explored the socio-ecological aspects of the disease. Our major assumptions include the presence of socio-ecological barriers to delivering high-quality mass drug administration leading to suboptimal treatments, barriers against improved hygiene and sanitation practices, and limited engagement of stakeholders. In this study, we explored factors that contribute to the persistence and recrudescence of trachoma in diverse population groups involving pastoralist and farming communities.

## Methods

**Research questions:** the study addresses two interrelated research questions, namely: 1) what are the major socio-ecological characteristics of districts characterized by persistent and recrudescent trachoma? 2) what are the factors associated with signs of trachoma in districts with persistent and recrudescent trachoma?

**Figure 1:**
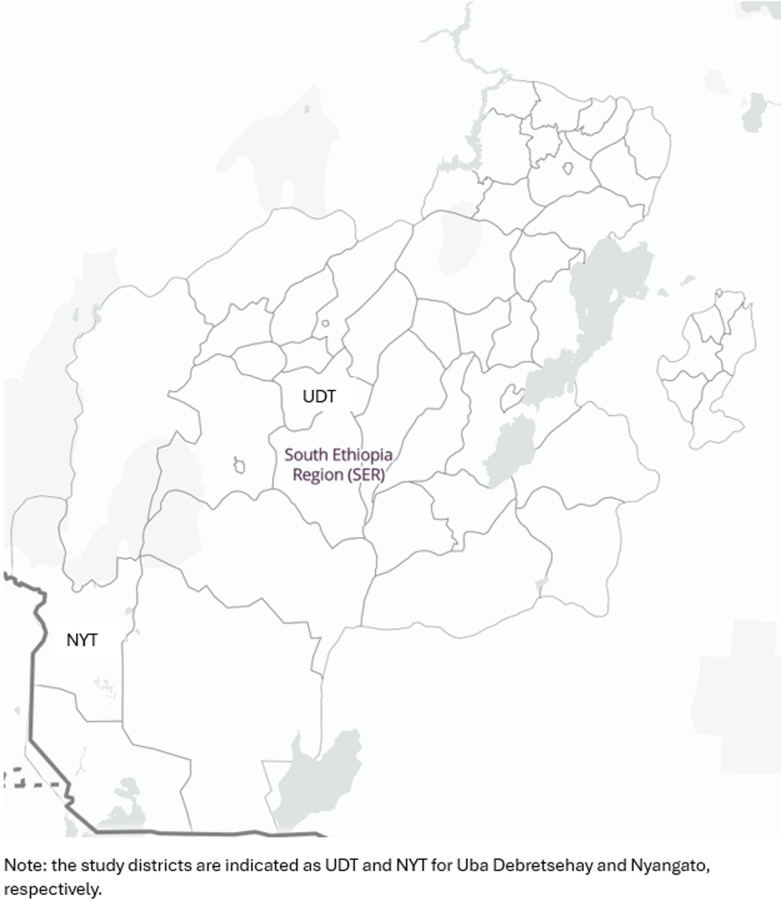
study area administration map

**Study setting:** the study was carried out in two districts of the Southern Region, Ethiopia. The study included a pastoralist and a predominantly farming district from Gofa Zone and South Omo Zone (Figure 1). Nyangatom and Uba Debretsehay districts are characterised by persistent and recrudescent trachoma, respectively. The challenge has been identified since 2021 standard surveys. SAFE intervention, especially the surgery and antibiotic component has been implemented in the two districts since 2017. During the study period, there were three persistent and 10 recrudescent districts in the two zones.

**Study design:** a community-based cross-sectional study design was employed. Study respondents were 18 years and older people living in a randomly selected household in the Nyangatom and Uba Debretsehay districts. The head of the household or another randomly selected adult (in the absence of the head) out of the list of adults living in the household was enrolled.

**Sample size:** the sample size for the study was determined using the formula for estimating the sample for a single population proportion for precision:


$$n=DEFF\times \left(\frac{z^{2}\times p\left(1-p\right)}{c^{2}}\right)\times e$$

Which is adapted from the Global Trachoma Mapping Project, a World Health Organization endorsed methodology [7]. Design effect (DEFF) of 2.63; 95% confidence interval (Z^2^) 1.96; expected prevalence (%) (p) is 4; absolute precision (%) (C^2^) is 2; non-response rate (e) of 1.2; final sample size (n) is 1.164; considering the presence of 1.5 adults per household (n=S/1.5), 776 heads of households or adult members of households per district were enrolled.

**Sampling technique:** a multistage sampling procedure was used to select study respondents. First, two zones were purposively selected namely with agrarian and pastoralist context. Second, from each zone, one district was randomly selected among three persistent and 10 recrudescent districts. Third, the list of all villages (clusters hereafter) was obtained from the respective health offices and used as a sampling frame. A total of 26 clusters and using compact segment sampling 30 households were selected. Finally, from each household, the head or a randomly selected adult member was enrolled in the absence of the head of the household. Households that have been residing in the area for less than a year were excluded. This is mainly due to their absence during the prior tropical data survey in the area that determined the status of the district-level prevalence.

**Data collection:** data were collected between May and October 2023. Data was collected by certified integrated eye care workers (IECWs) on trachoma disease identification and interventions, with at least a bachelor´s degree and a one-month additional training on primary eye care. These were given a two-day additional training on data collection tools. Daily field supervision was conducted by supervisors and principal investigator. Data was collected using interviewer-administered structured questionnaire in the Open Data Kit (ODK) system in Android devices. In addition to the tropical data questionnaire that mainly focuses on access to water and sanitation as well as trachoma grading, other major variables of interest include expanded socio-demographic information, treatment and treatment-related history, observations of facial cleanliness, fly density on the face of the respondent and children available at the time of the interview, access to primary eye care services and role of local leadership and their engagement.

**Data analysis:** data were downloaded from the server in Microsoft Excel and exported to SPSS 28 for analysis. Descriptive summaries were presented, described, and explained. Bivariate analysis was conducted in logistics regression for crude associations between the presence of trachoma and various independent variables individually. Factors with a p-value <0.25 in a bivariate logistic regression model, were included in the multivariable analysis. We used a multivariate logistic regression model to control various confounding factors in this study. In this study, we conducted the Hosmer and Lemeshow test to check model fitness, and we found with p-value greater than 0.05 (p-value=0.87) and confirmed the model fitness. We checked multicollinearity with variance inflation factor, considering variance inflation factor values less than 10.

**Ethical consideration:** ethical clearance was obtained from the University of South Africa, Research, and Ethics Committee (NHREC Registration number: Rec-240816-052 CREC Reference number: 45378932-CREC_CHS_2023). Each participant received a thorough explanation of the study's purpose, data to be collected, length of stay, potential risks, and benefits prior to providing informed consent. Participants had the option to withdraw and cease participation at any point during the data collection.

**Informed consent statement:** informed consent was obtained from all subjects involved in the study.

## Results

**Participant characteristics:** among the expected 1552 respondents, 1538 (99.1%) participated to the study. The age ranges between 20 and 95 and the mean (±SD) age was 33.42 years (±10.6) while the median was 30 years. Overall, 1186 (78%) of the respondents were between the age 20 and 39 years.

Generally, the study respondents are characterized by a low level of literacy with < two years average year of formal schooling, the mean (± SD) educational status of the respondents was 3.5 (±3.8) years (Table 1). This low level of schooling is partly attributable to large number of individuals who did not attend formal education. In terms of occupation, the majority of the study respondents are either engaged in mixed farming or agropastoral/pastoralism. Accordingly, 694 (44.8%) of the households rely on mixed farming while 682 (45.6%) of the respondents make their livelihoods from agro-pastoralism. Only 69 (4.5%) and 64 (4.2%) of the study respondents engage in formal employment and small business, respectively. Eleven respondents reported that their main occupations were housewives and students during the survey (Table 1).

**Table 1:** demographic characteristics of the study respondents in Southern Ethiopia, 2023

Variable	Category	Uba Debretsehay		Nyangatom		Total	
		Frequency	Percent	Frequency	Percent	Frequency	Percent
**Age category**							
	20-29 years	296	38.6	392	51.9	688	45.2
	30-39 years	269	35.1	230	30.4	499	32.8
	40-49 years	96	12.5	91	12.0	187	12.3
	50-59 years	62	8.1	27	3.6	89	5.8
	60 and above years	43	5.6	16	2.1	59	3.9
**Level of education**							
	No formal education	494	64.5	523	69.2	1017	66.8
	Grade 1-6-primary 1	179	23.4	162	21.4	341	22.4
	Grade 7-8-primary 2	34	4.4	29	3.8	63	4.1
	Grade 9-12-high school	59	7.7	42	5.6	101	6.6
**Major occupation**							
	Mixed farming	682	89.0%	0	0.0%	682	44.8%
	Agro-pastoralist or pastoralist	38	5.0%	656	86.8%	694	45.6%
	Formal employment	16	2.1%	53	7.0%	69	4.5%
	Small business	26	3.4%	38	5.0%	64	4.2%
	Religious service	2	0.3%	0	0.0%	2	0.1%
	Other	2	0.3%	9	1.2%	11	0.7%

**Household characteristics:** during the survey, 931 (74.7%) households reported that they keep their livestock separate to their main living house, while 315 (25.3%) do not. Generally, due to the herd size and pastoralist nature of the community, the respondents in Nyangatom reported presence of separate space for livestock compared to Uba Debretsehay. Smallholder farmers, tend to keep their small number of animals within the same shelter as humans.

### Water and sanitation-related characteristics

**Access to water:** accordingly, 946 (62.2%) of the households were using surface water (e.g. river, dam, lake, pond, stream, canal) in the dry season. Six hundred and sixty-seven (88.2%) of the respondents fetch surface water in Nyangatom compared to 279 (36.4%) of the respondents in Uba Debretsehay (Table 2). On average, households had to travel a round trip of about 52.3 (±92) minutes to fetch drinking water during the dry season. A district-desegregated analysis indicated that access to drinking water is challenging among the pastoralist communities of Nyangatom compared to Uba Debretsehay. Accordingly, the mean distance (±SD) traveled was 20.5 (±53) minutes in Uba Debretsehay compared to 85.9% (±92.1) in Nyangatom districts (Table 2).

**Table 2:** the situation of water, sanitation, and hygiene characteristics in Southern Ethiopia, 2023

Variable	Category	Uba Debretsehay		Nyangatom		Total	
		Frequency	Percent	Frequency	Percent	Frequency	Percent
**Main source of drinking-water**							
	Piped water into dwelling	73	9.5	8	1.1	81	5.3
	Piped water to compound/yard/plot	101	13.2	51	6.7	152	10.0
	Public tap/standpipe	66	8.6	2	0.3	68	4.5
	Tube well/borehole	58	7.6			58	3.8
	Protected dug well	105	13.7	1	0.1	106	7.0
	Surface water (e.g. river, dam, lake, pond, stream, canal)	279	36.4	667	88.2	946	62.2
Round trip time taken in the dry season	Time taken to collect drinking washing water (Mean (±SD))	20.47(±53.5)		85.91(±92.1)		52.26(±81.6)	
	Time taken to collect face washing water (mean (±SD)	25.2(±55.8)		84.64 (±85.4)		57.4(±79.0)	
**Main water sources used for washing faces**							
	Piped water into dwelling	74	9.7	8	1.1	82	5.4
	Piped water to compound/yard/plot	101	13.2	51	6.7	152	10.0
	Public tap/standpipe	57	7.4	1	0.1	58	3.8
	Tube well/borehole	56	7.3	1	0.1	56	3.7
	Protected dug well	94	12.3	1	0.1	95	6.2
	Surface water (e.g. river, dam, lake, pond, stream, canal)	316	41.3	668	88.4	984	64.7
**Latrine type**							
	Shared or public latrine	165	21.5	70	9.3	235	15.4
	Private latrine	553	72.2	198	26.2	751	49.3
	No structure, outside somewhere	44	5.7	485	64.2	529	34.8
	Other	4	0.5	3	0.4	7	0.5
**Defecate outside**							
	Yes	337	44.0	601	79.5	938	61.6
	Do not know	70	9.1	30	4.0	100	6.6
Handwashing facility (n=751)	Yes	364	65.8	89	44.9	453	60.3
Availability of water (n=453)	Yes	20	5.5	3	3.4	23	5.1
Availability of soap or detergent (n=453)	Yes	11	2.0	3	1.5	14	3.1

During the dry season, access to face washing water gets further challenging. As indicated in the following table, 984 (64.7%) of the respondents collect face-washing water from surface water sources. As indicated above the respondents pointed out that it takes over 25 minutes to access water for washing purpose with a standard deviation of 55 minutes. This is a significant time-intensive household chore (Table 2).

**Access to sanitation:** regarding access to sanitation, 235 (15.4%) and 751 (49.3%) of the respondents use either shared or private latrines, respectively. However, 529 (34.8%) of the respondents do not have any form of latrine, which implies open defecation. There is variation within the districts too. For instance, while 485 (64.2%) of the respondents in Nyangatom exercise open defecation, only 44 (5.7%) do so in Uba Debretsehay. In contrast, while 553 (72.2%) of the respondents use private latrines in Uba Debretsehay, only 198 (26.2%) of the respondents use the same in Nyangatom (Table 2).

This data is further validated by asking respondents about a community-wide practice of open defecation. Accordingly, the majority (61.6%) of the respondents believe that open defecation is practiced within their community (n=1522), while only 484 (31.8%) reported that their communities are open defecation-free. This ranges between 44% in Uba Debretsehay to 79.5% in Nyangatom. Disaggregated data at the district level also consistently confirms the pattern (Table 2).

**Hygiene practices among the respondents:** two hundred and ninety-eight (39.7%) of the households did not have a hand washing facility, and 430 (94.9%) did not have water, and 439 (96.9%) did not have soap, detergent, or other cleaning agent available at the handwashing facility, at the time of the visit/observation (Table 2). Sanitation services are scarcely present in Nyangatom compared to Uba Debretsehay. While 364 (65.8%) of the respondents have handwashing facility in Uba Debretsehay, only 89 (44.9%) of the respondents in Nyangatom own hand-washing facilities. Among households owning private latrines as well as handwashing facility, only 34 (6.1%) and 8 (4.0%) of them have handwashing facility around the latrines. Furthermore, only 11 (2.0%) and 3 (1.5%) of the households have soap, detergent, or other cleaning agents available at the handwashing facility, at the time of the visit/observation (Table 2).

Overall, 748 (49.1%) of the respondents reported unsafe disposal of child faeces including putting it into a drain or ditch, throwing it into the garbage, burying it, leaving it in the open, and other unsafe practices. Unsafe practices are reported by 205 (26.7%) and 543 (71.8%) of the respondents in Uba Debretsehay and Nyangatom districts, respectively.

**Environmental characteristics:** provided that a significant proportion of the households practice open defecation, during the survey, the data collection team made observations of the fly density within closer proximity. Only 194 (12.7%) of the households do not have observable flies; and few flies estimated at between 1-5 were observed in 383 (25.2%) of the households. Several flies, defined as more than 6 flies were observed in 911 (59.8%) households.

Flies are well-known vectors for transmission of the bacteria from an infected person and play a crucial role in increasing the risk of exposure among children. A large proportion of the observed households had flies in their environs. Data collectors observed one or more flies in the faces of 564 (42.5%) of the respondents during the interview. Generally, about 1-5 flies were observed on the face of large number of people observed in both districts. In contrast, flies were observed in the faces of one or more children from 704 (53%) households visited. Overall, there are more children with flies on their faces compared to adults in both districts.

**Facial cleanliness:** in this study in line with the global trachoma mapping project definitions, clean face refers to the face of an adult or child free from ocular and nasal discharge based on observation. Accordingly, an unclean face is characterized as the face of a child or an adult with observable dirt on the face, ocular discharge, nasal discharge, or flies [8]. Overall, in this study, children in 1075 (70.6%) of the study households had clean faces and in the remaining 447 (29.4%) households at least one or more children had unclean faces. It should be noted that accurate measurement of facial cleanliness has been a recognized challenge, despite the training provided [9]. Overall, 1201 (78.9%) of the respondents indicated that children´s face is washed less than once per day while 321 (21.1%) reported that they practice one or more times of face washing with water alone.

There are a large number of households that do not practice frequent face washing for their children in both districts. Overall, 1360 (89.4%) of the households reported that on average they wash their children´s faces less than once in a day with soap. In the meantime, it was found that about 162 (10.6%) of the households wash children´s face once or more using soap. Accordingly, the utilization of face washing using soap is not highly practiced among the households involved in this study.

**Clinical characteristics of the study households:** in this study, 1034 (67.7%) of the respondents indicated that there was no member of the family who had any of the six typical clinical symptoms of the disease. However, 492 (32.3% CI 30%, 34%) reported that there is at least one member of the family who has at least one of the six clinical manifestations of trachoma, which include, mild itching and irritation of the eyes and eyelids, eye discharge containing mucus or pus, eyelid swelling, light sensitivity (photophobia), red eye (redness), and eye pain. A district-by-district analysis indicated that there are variations in the proportion of households reporting at least one member of the household reporting the typical symptoms of infectious trachoma. Accordingly, about 187 (40.3%) and 305 (24.4%) of the households reported the presence of at least one member of the household manifesting one or more typical clinical symptoms of infectious trachoma during the survey.

**Factors associated with symptoms of trachoma infection:** in bivariate logistic regression analysis model, age, sex, respondent's education, wealth quantile, practice, a separated place for an animal, animal droppings in the compound, ever been informed about trachoma, household members received microdermabrasion (MDA) treatment, children ever swallow Zithromax suspension, availability of primary eye care facility, surgery correct eyelash touching eyeball, ever used eye drops in the past 3 years, respondent or respondent family members examined for eye, ever worried about/family gets trachoma, ever been worried about you/family members get blind due trachoma were the variables with statistically significant at a p-value of less than 0.25 and candidate for multivariate logistic regression.

However, after adjustments for possible confounders in the multivariable logistic regression analysis, wealth quantile, practice, separated place for an animal, ever been informed about trachoma, household members received MDA treatment, children ever swallow Zithromax suspension, availability of primary eye care facility, local (district) leaders´ engagement, observed faces, face washing with soap, ever worried about/family get trachoma, ever treated in the past three years, frequency of treatment received (how many times did you get treated), tablet doses received were independently associated with symptoms of trachoma infection at p-value less than 0.05.

Symptoms of trachoma were significantly associated with the presence of households in the poorest quantile (AOR=1.36, 95% CI:1.0,1.75), individuals not received MDA treatment in the last three years (AOR=2.8, 95% CI:1.5, 5.2, p<0.001), children not treated with Zithromax suspension (AOR=2.5,95% CI:1.5, 4.2, P<0.001), not ever treated in the past three years (AOR=11.2,95% CI: 6.5, 19.3, p<0.001), taken less than 3 dose of Zithromax (AOR=1.94,95% CI:1.32, 2.86, P<0.001), treated 0 (zero) times in the last 3 years (AOR=8.0,95% CI:2.4, 26.7, p<0.001) (Table 3).

**Table 3:** bivariate and multivariate analysis of factors associated with typical clinical symptoms of infectious trachoma in Southern Ethiopia, 2023

Variables	Category	Trachoma symptoms		X^2^ p-value	COR	AOR	P-value
		No	Yes				
**Wealth quantile**							
	Low	368	211	X^2^=7.1	1.4(0.54, 3.79)	1.36(1.0,1.75)**	0.019
	Middle	196	108	P=0.02	0.85(0.22,3.34)	1.4(1.0, 1.9)	0.027
	High	436	173		1	1	1
**Face washing with water (children)**							
	<Once per day	202	119	X^2^=4.18	1.3(1.0, 1.69)	1	1
	> Once per day	828	373	P=0.025	1	0.86 (0.58,1.27)	0.456
**Face washing with soap (children)**							
	Rarely	944	416	X^2^=17.6	0.49(0.36, 0.69)	1	1
	>once per day	86	76	P<0.001	1	0.59 (.36, 0.97)	.040
**Open defecation**							
	No	405	179	X^2^=1.22	0.88(0.70, 1.10)	1	1
	Yes	625	313	P=0.148	1	1.08 (0.78,150)	0.632
**Did everyone in this household received MDA/treatment?**							
	No	44	238	X^2^=440	22.4(15.7, 31.8)	2.8(1.5, 5.2)**	<0.001
	Yes	948	229	P=<0.001	1	1	
**Children ever swallow zithromax suspension**							
	No	86	146	X^2^=117	4.6(3.45 6.21)	2.5(1.5, 4.2)**	<0.001
	Yes	944	346	P=<0.001	1	1	
**Have you and members of the household been ever treated in the past three years?**							
	No	39	288	X^2^=591.6	35.8(24.8, 51.76)	11.2(6.5, 19.3)	P<001
	Yes	991	204	P<0.001	1	1	
**In the last 3 years, how many times/rounds did you and the family get treated?**							
	0	43	239	X^2^=489.9	32.01(21.6, 47.30)	8.0(2.4, 26.7)	<0.001
	1	74	66	P<0.001	5.1(3.45, 7.63)	6.0(3.8, 9.4)	<0.001
	2	360	91		1.45(1.06, 1.99)	1.9(1.3, 2.8)	<0.001
	3	553	96		1	1	
**Doses received by each member of the household**							
	Less than 3 tab	223	296	X^2^=219.7	5.45(4.32, 6.90)*	1.94(1.32, 2.86)	<0.001
	Greater than 3 tab	807	196	P<0.001	1	1	
**Local/district leaders’ engagement**							
	No	779	5	X^2^=5.7	0.66(0.50, 0.86)	1	1
	Yes	723	15	P=0.014	1	0.71 (.52,.99)	0.04

1= reference; ** significate at a p-value <0.001 and * significant at a p-value <0.05 level; AOR: adjusted odds ratio; COR: crude odds ratio; MDA: microdermabrasion

## Discussion

As indicated in the latest premier by leading researchers globally, the interpretation of clinical symptoms of trachoma is highly influenced by contexts [3]. For instance, in trachoma, hyperendemic communities such as Uba Debretsehay and Nyangatom, conjunctival infections, the relative economic status of the patient mainly being from the poorest segment of the community, and impression of the patient´s personal and family hygiene will lead to a reasonable conclusion that the disease manifestations could be attributable to active trachoma. In this study about 32.3% of the study households reported the presence of at least one member of the household with one or more symptoms of active trachoma. This finding is comparable to the latest systematic review which reported 26.9% prevalence of active trachoma among children in Ethiopia and the estimate for Southern Nations, Nationalities, and Peoples' Region (SNNP) was about 36% [10]. Another study conducted in an adjacent zone to the Uba Debretsehay district reported that the prevalence of active trachoma was about 37.9% of children aged 1-9 years have active trachoma in the study area [11]. Latest studies reported 25.43% and 37.5% prevalence of active trachoma in Southern Nations, Nationalities, and Peoples' Region (SNNPRS) and Oromia, respectively [6]. Another study [12] reported a 35% prevalence after decades of SAFE implementation in Amhara.

Previous studies indicated an association between increased risk of trachoma amongst populations living in poorer households, communities, and continents [3]. In this study, the odds of manifesting typical clinical symptoms of infectious trachoma among the respondent households was 1.36 times (AOR=1.36) higher among poorer and middle-wealth quantile households compared to wealthier households. The trend analysis also showed chi-squared (X^2^=7.1, p=0.02) a significant association between poverty and the risk of manifesting typical clinical symptoms of trachoma infection. This is consistent with results from Northern Ethiopia, among war-affected communities [13]. Generally, these findings are consistent with previous studies which indicated the association between poverty and increased risk of trachoma [3,14,15].

Face washing has been a pillar of the WHO strategies for the effective elimination of trachoma as a public health problem and has been implemented across trachoma programs all over the endemic countries [3]. Respondents with children who had a face washing practice with soap were 41% less likely (AOR=0.59, p=0.04) to develop typical clinical symptoms of infectious trachoma when compared with their counterparts. Our findings are in line with other studies that investigated the importance of face washing with soap and found a significant association [16]. Unclean faces are associated with non-use of soap for face and hand washing and increased risk of active Trachoma [3,10,17].

There is evolving evidence as well as practice for more frequent than annual MDA. While some other studies indicated substandard treatment below 80%, in this study the population coverage is above the WHO threshold, and households with at least one treatment over the past three years were less likely to report typical symptoms of trachoma. Accordingly, the presence of household members (at least one person missing) who had not received Zithromax mass drug treatment in the last MDA (AOR=2.8, p<0.001) were 2.8 times higher risk of reporting typical symptoms of active trachoma.

The effectiveness of azithromycin for mass drug administration has been established since 1999 [18,19]. In this study, we confirmed that the treated population is by far in a better position despite the persistence of trachoma within the communities. A study from Northern Ethiopia reported 8.9 (AOR=8.9, p=0.01) increased risk for active trachoma among individuals who do not receive azithromycin [13]. A recent systematic review reported that a single dose of azithromycin showed a significant reduction of ocular infection [20].

Ensuring greater than 80% therapeutic coverage during mass drug administration is the recommended standard practice, meaning over 80% of the population should be treated with azithromycin and tetracycline [3]. Unlike various other studies [21-23], this study indicated about 80% of the population has been treated in the last MDAs, meeting the WHO treatment threshold. Achieving 80% treatment has been reported by various authors in earlier studies [24,25]. In practice, while the 80% coverage is acceptable, to ensure that the concept of herd immunity is achieved, it would have been much more meaningful for programs to understand the missing groups. However, there are limited studies that identified who the missing 20% of the population are. Overall, among households that precisely remember and reported a presence of at least one member of the household missing a treatment in the past three years, 62 (59%) reported that the missing members were children aged below 10 years, followed by children aged 10-15 years (32.4%).

In this study, we identified the predominantly missing group of the target population as well as indications for both random and systematic missing. Based on the analysis, both systematic and random omission of clusters and households has been observed. For instance, 11 out of 52 clusters contributed to 55% of the households that reported receiving no MDA in the past three years. This includes clusters with more than 1/3^rd^ of the respondents who reported missing MDA. Moreover, in 5 clusters over 63% of the respondents reported no MDA, and one cluster was generally omitted. In these five clusters, about 50% of the population reported missing MDA.

Similarly, children who did not swallow Zithromax suspension over the past three years had a 2.5 higher risk of developing trachoma infection when compared with those children who had swallowed Zithromax suspension (AOR=2.5, p<0.001). This is a novel finding in the field established by this data. However, this evidence does not support or oppose targeted treatment for children, instead reinforces the reach for universal coverage and reaching children during MDA.

The risk of exposure further grows with a lack of treatment for three consecutive years, often a good indicator of proper completion of the recommended dose on average. The data indicated that the odds of trachoma infection were 11.2 times higher among those who were not ever treated in the past three years when compared with counterparts (AOR=11.2, p<0.001) to the extent of researchers´ knowledge, there are no previous studies that established the impact of missing one or more rounds of treatment. Moreover, the odds of developing symptoms of trachoma infection were 1.94 times higher among those who had taken less than 3 tablets when compared with greater than 3 tabs dose (AOR=1.94, P<0.001). Respondents who had been treated 0 (zero) times in the last 3 years had an 8.0 times higher risk of developing trachoma infection when compared with their counterparts (AOR=8.0, p<0.001).

The role of leadership and governance has never been investigated in trachoma studies. To our knowledge, this study is the first to shine a light on the effective engagement of local leadership. The district leaders who had an engagement in trachoma prevention control program in the communities were 21% (AOR=0.71, p=0.044) less likely to develop typical clinical symptoms of infectious Trachoma when compared with those who had no engagement in trachoma program control.

**Limitations of the study:** due to the nature of the study design may not establish a cause-effect relationship. Moreover, we did not conduct clinical investigation and laboratory diagnosis to confirm infection, mainly due to our interest in designing alternative community-based strategies to address persistence and recrudescence in a community setting. This limitation has an inherent strength for establishing a community-based surveillance in persistent districts that overcomes the limitations of the other two namely the tropical data systems and serology and tropical data systems. Furthermore, objective measurement of environmental factors and facial cleanliness is a continued challenge.

## Conclusion

The presence of a large proportion of study participants manifesting typical symptoms of trachoma further re-affirms the continued persistence of the disease within the study communities. Generally, both districts are characterized by segments of the population missing optimum treatment as well as poor hygiene and sanitation practices. The most often missed population groups are children aged less than 10 years who are often known as hotspots for transmission. Our evidence supports the importance of adhering to optimal treatment guidelines, leaving no one behind, and the need for adequate treatment coverage.

### What is known about this topic

- *The issue of persistent and recrudescent trachoma came to prominence among the trachoma control stakeholders since 2021; the urgency of generating evidence has been the foremost recommendation;*
- *The presence of continued transmission of trachoma despite the implementation of the SAFE strategy has been recognized, but with very limited evidence;*
- *More momentum on evidence generation has been set by multiple research grants to investigate the persistence and recrudescence of trachoma as of 2024.*

### What this study adds

- *This study is one of the pioneer pieces of evidence addressing the persistence and recrudescence of trachoma; we identified that there are segments of populations that are not reached through MDA for various reasons which include never-treated and sub-optimally treated population groups;*
- *In this study we confirmed that the treated population is by far in a better of position despite the persistence of trachoma within the communities;*
- *The study provided an alternative approach for establishing a locally managed surveillance system, in conjunction with existing survey approaches; it is a pioneer reporting the role of local leadership to accelerate elimination.*
